# NR4A1 inhibition synergizes with ibrutinib in killing mantle cell lymphoma cells

**DOI:** 10.1038/s41408-017-0005-z

**Published:** 2017-11-23

**Authors:** Yangguang Li, Fangyu Wang, Li Lu, Fen Zhu, Shengjian Huang, Krystle Nomie, Liang Zhang, David T. Yang, Wei Huang, Brad S. Kahl, Stephen Safe, Michael Wang, Lixin Rui

**Affiliations:** 1 Department of Medicine, University of Wisconsin School of Medicine and Public Health, Madison, WI 53792 USA; 2 Carbone Cancer Center, University of Wisconsin School of Medicine and Public Health, Madison, WI 53792 USA; 3 Department of Lymphoma and Myeloma, The University of Texas MD Anderson Cancer Center, Houston, TX 77030 USA; 4 Department of Pathology and Laboratory Medicine, University of Wisconsin School of Medicine and Public Health, Madison, WI 53792 USA; 5 Department of Medicine, Washington University School of Medicine, St. Louis, MO 63110 USA; 6 Department of Veterinary Physiology and Pharmacology, Texas A&M University, College Station, TX 77843 USA

NR4A1 (Nur77, TR3, NGFI-B), a member of the nuclear receptor family, is known as an immediate early or stress response gene^1^. NR4A1 plays a physiological role in development and cellular homeostasis, and is also involved in tumorigenesis^1^. NR4A1 is overexpressed and exhibits oncogenic activity in many solid cancers, whereas it acts as a tumor suppressor in hematologic malignancies^1^. This is likely due to a dual role for NR4A1 in mediating cell proliferation/survival vs apoptosis. As a transcription factor, NR4A1 is primarily localized in the nucleus and regulates gene expression to enhance cell proliferation and survival^2^. When NR4A1 is exported from the nucleus to mitochondria, it binds BCL-2 and subsequently induces cell apoptosis^3,4^.

NR4A1 was first characterized as a tumor suppressor in a report showing the rapid development of acute myeloid leukemia in NR4A1 and NR4A3 double knockout mice but not in single knockout animals^5^. Reduced expression of these two genes is a common feature in human acute myeloid leukemia cells^5^. In diffuse large B-cell lymphoma and high-grade follicular lymphoma, low NR4A1 expression was significantly associated with a non-germinal center B-cell subtype and with poor overall survival^6^. NR4A1 overexpression induced apoptosis of diffuse large B-cell lymphoma cells and inhibited xenografted tumor growth^6^. These findings together with our recent study^7^ prompted us to investigate the role of NR4A1 in mantle cell lymphoma (MCL), a currently incurable non-Hodgkin lymphoma. Our RNA sequencing (RNA-seq) analysis in four MCL cell lines identified that NR4A1 is one of six common downregulated genes by Bruton tyrosine kinase (BTK) short hairpin RNA^7^ (Fig. 1a). This result was further confirmed by quantitative PCR (Supplementary Fig. 1A). BTK is a key component of the early B-cell antigen receptor (BCR) signaling pathway and its inhibitor ibrutinib has emerged as an effective therapeutic agent for the treatment of MCL^8^. To further verify that NR4A1 is a target gene of BCR/BTK signaling, we stimulated naive B cells from peripheral blood with α-IgM or combined with CD40L. Indeed, both messenger RNA and protein levels of NR4A1 were significantly increased with the BCR stimulation (Supplementary Fig. 1B, C), consistent with a previous study^9^.

**Fig. 1:**
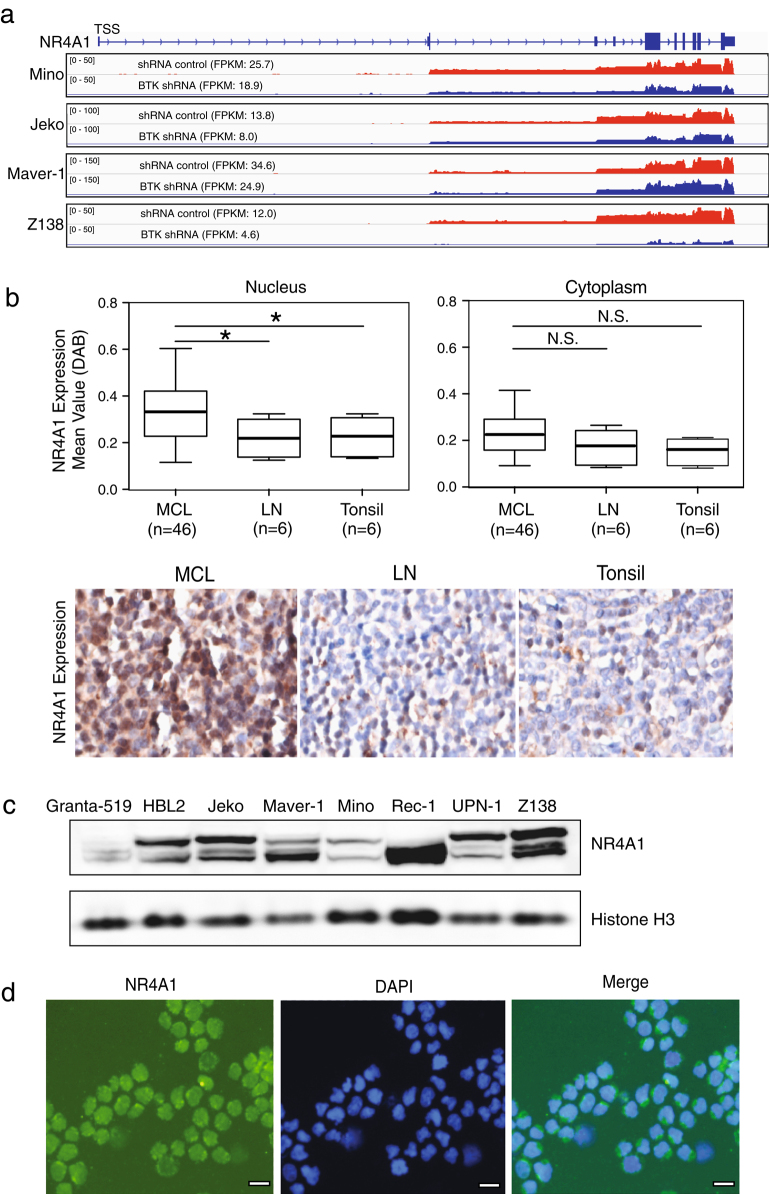
NR4A1 expression in MCL patients and cell lines **a** Reduced NR4A1 expression at a transcriptional level by RNA-seq after 2 days of BTK knockdown by shRNA in four indicated cell lines. RNA-seq data are shown as read density tracks. **b** A tissue microarray (TMA) containing 46 cases of MCL and 6 each of normal lymph nodes and tonsils was used for immunohistochemical staining for NR4A1 expression and analyzed with InformTM advanced image analysis software. Representative staining images are shown. **c** Immunoblot assay for expression of NR4A1 in eight MCL cell lines. Histone H3 served as a loading control. **d** Immunofluorescence staining for NR4A1 expression and localization in Rec-1 cells. Scale bar, 10 μm. *FPKM* fragments per kilobase of exon per million fragments mapped

Next, we determined NR4A1 expression in a tissue microarray of 46 MCL cases by immunohistochemical (IHC) analysis. All these MCL cases bore the typical chromosomal translocation *t*(11:14) involving cyclin D1 with the morphological characteristics of MCL, as described previously^10^. As shown in Fig. 1b, NR4A1 was mainly localized in the nucleus and the average of nuclear NR4A1 protein expression in MCL cases was significantly higher than that in normal B cells from tonsils or lymph nodes. NR4A1 protein was rarely present in the cytoplasm of both MCL and normal B cells. Also, NR4A1 expression with two main isoforms was detected by immunoblot analysis in all eight MCL cell lines tested, despite at various levels (Fig. 1c). Consistent with the above IHC analysis, immunofluorescence staining in Rec-1 cells displayed nuclear localization of NR4A1 (Fig. 1d).

Given that NR4A1 is expressed in MCL, mainly present in the nucleus, and is induced for expression by BCR/BTK signaling, we hypothesized that NR4A1 is not a tumor suppressor but rather a potential oncogene in MCL. To test this hypothesis, we first expressed NR4A1 complementary DNA using an inducible retroviral vector in Mino and Granta-519 cell lines, both of which have relatively low levels of endogenous NR4A1 expression. We confirmed NR4A1 expression by both immunoblot and immunofluorescence analysis (Supplementary Fig. 2A, B). After 6 days of induction, NR4A1 expression did not affect cell growth in the culture of Mino and Granta-519 cells (Supplementary Fig. 2C, data not shown). Then, we verified this result using the NR4A1 agonist cytosporone B (Csn B). Although Csn B treatment significantly increased NR4A1 expression in Mino and Jeko cells, the cell viability and cell cycle progression were not changed (data not shown, Supplementary Fig. 2C). These data suggest that NR4A1 does not have tumor suppressor-like activity in MCL.

Since NR4A1 is a target gene of BTK, we tested whether their expression levels are correlated in MCL. On the basis of our recent work demonstrating high levels of BTK expression in the majority of the MCL cases^7^ and the above NR4A1 expression result, we indeed found a strong positive correlation between BTK and NR4A1 (*p* < 0.0001) (Fig. 2a). This result led us to perform functional analysis on NR4A1. We used an inducible CRISPR/Cas9 system to knock out NR4A1 by single-guide RNA (sgRNA) in Jeko and Rec-1, two NR4A1 highly expressed cell lines. Immunoblot analysis identified two single cell clones that showed a high efficiency of knockout after 6 days of sgRNA induction (Fig. 2b). Although expression of these NR4A1 sgRNAs alone did not reduce cell viability (Supplementary Fig. 3A), their expression significantly enhanced the BTK inhibitor ibrutinib-mediated cytotoxicity to the cultured MCL cells (Fig. 2b). We confirmed this result with NR4A1 antagonist DIM-C-pPhOH^11^, which also synergized with ibrutinib in killing the cells (Fig. 2c, Supplementary Fig. 3B, C). Reduced cell viability by co-treatment of DIM-C-pPhOH and ibrutinib is due to reduced cell proliferation and increased apoptosis, based on cell cycle analysis (Fig. 2d). Of note, DIM-C-pPhOH significantly reduced cell viability of primary MCL cells from all seven patients examined despite a short period (24 h) of treatment (left, Fig. 2e). More importantly, the toxic effect of DIM-C-pPhOH treatment was observed on three primary ibrutinib-resistant patient samples. Furthermore, DIM-C-pPhOH enhanced or restored ibrutinib-mediated cell killing significantly in an ibrutinib-sensitive or ibrutinib-resistant patient sample (right, Fig. 2e) and a similar trend was also observed in the rest of patient samples (data not shown). Taken together, these results suggest that NR4A1 is a potential oncogene in MCL.

**Fig. 2:**
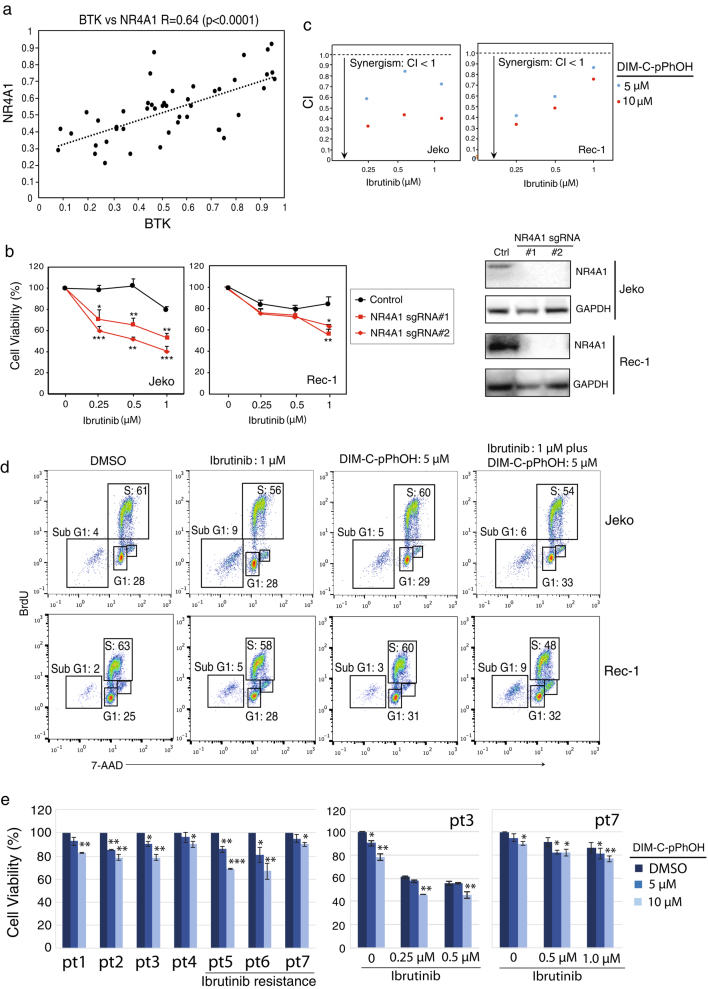
NR4A1 inhibition synergizes with ibrutinib in killing MCL cells **a** Shown is a strong positive correlation between NR4A1 and BTK in 46 MCL cases analyzed by Prism. “*r*” denotes non-parametric Spearman’s rank correlation coefficient. **b** Cell viability analysis after induction of NR4A1 sgRNA expression with different doses of ibrutinib in Jeko and Rec-1 cells. Cells were treated with doxycycline to induce NR4A1 sgRNA expression, and then incubated with ibrutinib for 6 days before trypan blue dye exclusion viability assay. The CRISPR/cas9 empty vector served as a control. Error bars represent mean ± SD of triplicates (*p < 0.05, **p < 0.01, ***p < 0.001). NR4A1 knockout by sgRNA was confirmed by immunoblot assay. **c** Synergism between DIM-C-pPhOH and ibrutinib in cell killing in Jeko and Rec-1 cells. Cells were treated with ibrutinib and DIM-C-pPhOH 6 days before trypan blue dye exclusion viability assay. Combination index (CI) was calculated with CompuSyn software. **d** Cell cycle was analyzed by BrdU and 7-AAD double staining after treatment with the single drug or combination for 6 days. **e** Ex vivo cell viability assay of primary cancer cells from seven MCL patients including three ibrutinib-resistant samples. The cancer cells were incubated with the indicated concentrations of DIM-C-pPhOH alone (left) or combined with 0.25 or 0.5 μM of ibrutinib (right) for 24 h before CellTiter-Glo luminescent cell viability assay. Error bars represent mean ± SD of triplicates (*p < 0.05, **p < 0.01, ***p < 0.001)

To investigate the molecular mechanisms underlying the oncogenic activity of NR4A1, we performed the whole-genome transcriptome analysis in NR4A1 knockout cell lines by RNA-seq (Supplementary Table 1). As expected, we found that a significant number of genes were upregulated and downregulated in either Jeko or Rec-1 cells after NR4A1 knockout (Supplementary Fig. 4A). Among these upregulated and downregulated genes, ~1300 genes were overlapped between Jeko and Rec-1 cells. We used gene set enrichment analysis to identify the signaling pathways these upregulated and downregulated genes involve. The results revealed significant enrichment in gene signature of the cell cycle G2M checkpoint and PI3K/AKT/mTOR signaling pathways in both cell lines (Supplementary Fig. 4B), consistent with our recent observations in several solid cancers^12–15^. Of note, cell cycle-related genes such as CDK2, CDK4, and E2F1 were downregulated, whereas genes in the PI3K pathway including PTEN, PTPN11, and GSK3B were upregulated (Supplementary Fig. 4B). RNA-seq analysis in our recent study has demonstrated that the common enriched signature upon BTK inhibition in MCL cells is associated with regulation of apoptosis^7^. Consistent with this finding, we observed increased apoptotic population (sub G1) when Jeko and Rec-1 cells were treated with ibrutinib (Fig. 2d). These RNA-seq analyses and functional assays provide the molecular mechanisms by which NR4A1 inhibition synergizes with ibrutinib through targeting differential signaling pathways.

In summary, we show here that the BCR/BTK target gene NR4A1 is a potential oncogene in MCL, rather than a tumor suppressor as reported in several other hematological malignancies. NR4A1 is highly expressed in the majority of MCL cases and also positively correlated in expression with BTK. More importantly, genetic and pharmacological inhibition of NR4A1 enhances the cytotoxicity mediated by the BTK inhibitor ibrutinib. RNA-seq analysis elucidated that NR4A1 inhibition changes expression of signature genes in the cell cycle G2M checkpoint and PI3K/AKT/mTOR signaling pathways, which are associated with cell proliferation. Our findings suggest that NR4A1 is a novel promising molecular target in MCL. Although recent clinical investigation of ibrutinib in MCL revealed frequent responses, most responses were not long lasting^8^, suggesting that combinations of targeted agents that inhibit distinct oncogenic pathways will be necessary. The study provides the mechanistic rationale for co-targeting of BTK and NR4A1 as a new therapeutic strategy for MCL, especially for patients with primary resistance to ibrutinib.

## Electronic supplementary material

SUPPLEMENTAL MATERIAL

Supplementary Table 1
