# Supplementary material for: NR4A1 inhibition synergizes with ibrutinib in killing mantle cell lymphoma cells
Source: Blood Cancer J. 2017 Nov 23;7(12):632. doi: 10.1038/s41408-017-0005-z (PMC5802686; doi:10.1038/s41408-017-0005-z)
Supplement: Supplementary file 1 — SUPPLEMENTAL MATERIAL [file 41408_2017_5_MOESM1_ESM.pdf]

Supplementary Information

NR4A1 Inhibition Synergizes with Ibrutinib in Killing Mantle Cell Lymphoma Cells

Yangguang Li[1,2], Fangyu Wang[1,2], Li Lu[1,2], Fen Zhu[1,2], Shengjian Huang[3], Krystle Nomie[3], Liang Zhang[3], David T. Yang[2,4], Wei Huang[4], Brad S. Kahl[5], Stephen Safe[6], Michael Wang[3] and Lixin Rui[1,2#]

[1]Department of Medicine and [2]Carbone Cancer Center, University of Wisconsin School of Medicine and Public Health, Madison, WI 53792, USA; [3]Department of Lymphoma and Myeloma, The University of Texas MD Anderson Cancer Center, Houston, TX 77030, USA; [4]Department of Pathology and Laboratory Medicine, University of Wisconsin School of Medicine and Public Health, Madison, WI 53792, USA; [5]Department of Medicine, Washington University School of Medicine, St. Louis, MO 63110, USA; [6]Department of Veterinary Physiology and Pharmacology, Texas A&M University, College Station, Texas, TX 77843, USA.

[#]Corresponding author: lrui@medicine.wisc.edu.

Materials and Methods

Cell culture and reagents.

All MCL cell lines were grown in RPMI 1640 media (Hyclone) supplemented with 20% FBS (fetal bovine serum, Atlanta Bilogicals), 2 mM GlutaGROTM (Corning Cellgro), 100 U/ml penicillin, 100 μg/ml streptomycin (Corning Cellgro), 1×MEM-NEAA (Quanlity Biological, Inc.), and 1 mM Sodium Pyruvate Solution (Hyclone). All cultures were routinely tested for mycoplasma contamination. All cells were cultured in a 5% CO2 atmosphere at 37°C. Cytosporone B (Csn B), ibrutinib (Selleck Chemical LLC (TX, USA)), DIM-C-pPhOH (kindly provided by Prof. Stephen Safe, University of Texas) were dissolved in DMSO in a stock solution of 10 mM and stored at -20°C.

Immunohistochemical staining and quantification.

A tissue microarray (TMA) of 46 cases of mantle cell lymphoma was obtained from our previous study (Oberley MJ, *et al. Histopathology* 2013), with approval from the University of Wisconsin-Madison institutional review boards (UW protocol M-2008-1011 and MCRF protocol SHA 10109). The protein expression level was analyzed with the antibodies NR4A1 (Abcam, #ab48789) and BTK (Cell SignalingTechnology, #8547). Protein expression was scored using InformTM advanced image analysis software. Scores of triplicate cores were averaged for each case.

Primary cancer cell culture and viability assay.

Peripheral blood, apheresis, biopsy tissues isolated from spleen and lymph nodes, bone marrow aspirates, ascites, or pleural effusion were obtained from mantle cell lymphoma patients who provided informed consent. The sample collection protocol was approved by the Institutional Review Board at

The University of Texas MD Anderson Cancer Center. All procedures were conducted in accordance with the Declaration of Helsinki. Mononuclear cells were separated by Ficoll-Hypaque density centrifugation, and tumor cells were isolated using anti-CD19 antibody-coated magnetic microbeads (Miltenyi Biotec, Auburn, CA, USA). Next, the isolated cells were frozen by reducing the temperature at approximately 1°C per minute using a cryo-freezing container (ThermoScientific) and placed at -80°C until use. To thaw the cells, the crovials containing the frozen cells were removed from the freezer and placed immediately into a 37°C water bath with gentle swirling to thaw the cells. Next, the vials were transferred into a laminar flow hood, and pre-warmed RPMI-1640 medium (Life Technologies, Grand Island, NY, USA) supplemented with 10% heat-inactivated fetal bovine serum, penicillin (10,000 units/mL, Sigma, St. Louis, MO, USA), streptomycin (10 mg/mL, Sigma), and L-glutamine (29.2 mg/mL, Life Technologies). The cells were then centrifuged at approximately 300 × g for 5 minutes, the supernatant was decanted, and the cells were resuspended in growth medium before the cell viability assay was performed.

Cell viability was measured using the CellTiter-Glo Luminescent Cell Viability Assay (Promega, Madison, Wisconsin, USA). Cells were seeded in triplicate in a 96-well plate with 2 x 10^5 cells per well and were treated with DIM-C-pPhOH alone or combined with ibrutinib for 24 hours
In the last 30 minutes, 50 µL of CellTiter 96 Aqueous One Solution Reagent were added to the culture wells and incubated at 37oC in 5% CO2. Light absorbance of formazan was measured at 495 nm on a universal microplate reader equipped with KC4 software (Biotek Instruments, Winooski, Vermont, USA).

Plasmids construction and inducible CRISPR/Cas9 system.
For the inducible system, the pR-CMV-Cas9-2A-Hygro (Cellecta, Inc, USA) was used for Cas9 expression, and pRSIG16-U6Tet-(sg)-CMV-TetRep-2A-TagRFP-2A-Puro (Cellecta, Inc, USA) was used for sgRNA expression, with the NR4A1 sgRNA sequence TGATGAACTCAGGGGTCAGG. After generating stable cell lines expressing Cas9 with hygromycin selection, single cell clones were obtained. The clones with high levels of Cas9 expression were used for a secondary infection with sgRNA virus targeting NR4A1. The sgRNA expressing cells were selected with puromycin. Single cell clones were sorted by flow (BD FACSAria with Biosafety Cabinet), and the knockout efficiency was verified by immunoblot after doxycycline induction.

Cell line viability assay.
Cell viability was measured after DIM-C-pPhOH or ibrutinib treatment with an antomatic cell counter according to the manufacture's instruction. Briefly, cells were harvested and suspended, and mixed with equal volume of 0.4% trypan blue. Ten µl of cell suspension was loaded onto TC20 system (Bio-Rad) counting slides, and the number of viable cells was quantified on a TC20 automated cell counter (Bio-Rad).

Immunoblot assay.
Cells were lysed using MAPK lysis buffer (4 mM sodium pyrophosphate, 50 mM HEPES, 100 mM NaCl, 10 mM EDTA, 10 mM sodium floride, 2 mM rothovanadate, pH 7.5) with protease inhibitor cocktail (sigma). Protein concentrations were determined by BCA assay (Thermo Scientific). Proteins were separated by SDS-PAGE and transferred to nitrocellulose membrane. After blocking with non-fat milk, the membrane was probed with a primary antibody. The primary antibodies included NR4A1 (Cell Signaling Technology, #3960), GAPDH (cell signaling technology, #3683), and histone H3

(Abcam, #ab1791). After blotting with the HRP-conjugated secondary antibody, the membrane was developed using chemiluminescence HRP substrate kit (Thermo Scientific).

Cell cycle and apoptosis analysis (BrdU/7-AAD).

Cell cycle was analyzed with the FITC BrdU Kit (BD Pharmingen, San Diego, CA, USA), following manufacturer's instructions. Cells were incubated with 10 µM DIM-C-pPhOH for 72 h, and then pulsed with BrdU for 12h at 37°C. The cells were washed in the staining buffer, fixed/permeabilized with the Cytofix/Cytoperm buffer and washed with the Perm/Wash buffer. After permeabilization, cells were treated with DNase for 1 h at 37°C, and then stained with FITC-conjugated anti-BrdU antibody and 7-AAD (25 µg/ml) before flow cytometric analysis. DNA contents were analyzed using FlowJo software.

Quantitative RT-PCR (qPCR).

Total RNA was extracted using RNeasy Plus Mini Kit (Qiagen) according to the manufacture's protocol, and quantified with Nanodrop lite spectrophotometer
(Thermo Scientific). For each sample, 1 µg RNA was reverse transcribed with a first-strand cDNA synthesis kit (Invitrogen). qPCR was performed using an ABI Stepone Plus Real-Time PCR System and SYBR Green mix (Applied Biosystems). Primers for qPCR, GAPDH Forward, 5'-GGGAAACTGTGGCGTGAT-3', Reverse, 5′-GAGTGGGTGTCGCTGTTGA-3′; NR4A1 Forward, 5′-GCAAGTGGGCGGAGAAGAT-3′, Reverse,5′-CCTCGCCTGGCTTAGACCT-3'. Data were normalized to GAPDH.

Naïve B cell isolation.

Naïve B cells were collected from peripheral blood mononuclear cells (PBMC), with approval from the University of Wisconsin-Madison institutional review boards (#2013-1570). Naïve B cells were isolated using the isolation kit (Miltenyi Biotec #130-091-150) according to the manufacturer's instructions. Briefly, PBMC was isolated by density gradient centrifugation, and blood cells were lysed with ACK lysing buffer. Naïve B cell biotin-antibody cocktail was then added and incubated for 5 min at 4 °C. Then, anti-biotin microbeads were added and incubated for additional 10 min at 4 °C. Negative selection was performed by MACS Pro machine. The purity was measured by FITC-CD20 (clone: LT20, Miltenyi Biotech) and PE-CD27 (clone O323, ebiosciences) staining (~98%).

Immunofluorescence staining.

Rec-1 or Naïve B cells were plated and fixed with 4% paraformaldehyde. Unspecific bindings were blocked with 5% fetal bovine serum for 60 min prior to the incubation with NR4A1 antibody (Cell Signaling Technology, #3960). Cells were subsequently washed three times and further incubated with RP-conjugated anti-mouse secondary antibody for 0.5 h and then stained with 5 ng/ml DAPI to visualize the nucleus for 5 min. The results were analyzed using a Nikon A1 confocal microscope.

RNA-seq.

RNA-seq analysis was performed as described previously (Li Y, *et al. Oncogene* 2016). Briefly, two sgNR4A1 (#1 and #2) in Jeko and Rec-1 cells were induced for expression with 20 ng/ml of doxycycline for 2 days and non-induced cells served as a control. Total RNA from cells was extracted by RNeasy plus mini kit (Qiagen, Valencia, CA, USA). RNA-seq libraries were prepared using Illumina Truseq stranded mRNA LT sample preparation kit (San Diego, CA, USA) according to the manufacturer's protocol. After sequencing, the coverage of 20-40 million reads was obtained for each

cell line. Reads were mapped to the human genome (hg19) using TopHat (v2.0.8b). The differential expressed transcript level between samples was calculated by Cuffdiff (v2.1.1) with the provision of a GTF annotation file (hg19). Heatmap was produced using R program. RNA-seq data are available at http://www.ncbi.nlm.nih.gov/geo/ (accession #GSE106092).

Gene set enrichment analysis was conducted using the GSEA software (http://software.broadinstitute.org/gsea/index.jsp; Subramanian A, *et al. Proc Natl Acad Sci USA*, 2005), and “hallmark gene sets (h.all.v5.2.symbols.gmt)” and “GO biological processes (c5.bp.v5.2.symbols.gmt)” were based on the molecular signatures database.

Supplementary Figure Legends

Supplementary Figure 1. (A) The NR4A1 mRNA change was validated by qPCR after 2 days of BTK knockdown by shRNA. Error bars represent mean ± SD of triplicates. (B) Naïve B cells were obtained from PBMC, NR4A1 expression was then measured after stimulation with anti-IgM/CD40L or both for 6 h and 24 h by qPCR. Error bars represent mean ± SD of triplicates. (C) Immunofluorescence staining of NR4A1 in naïve B cells was performed after 24 h of anti-IgM stimulation.

Supplementary Figure 2. (A) After 3 days of doxycycline induction, NR4A1 expression in Mino cells was confirmed by immunoblot analysis. (B) Immunofluorescence analysis shows NR4A1 overexpression and its nuclear localization. (C) Mino control and NR4A1 expressing cells were treated with/without doxycycline for 6 days before trypan blue dye exclusion viability assay. Error bars represent mean ± SD of triplicates. (D) NR4A1 expression in Mino and Jeko cells treated with 10 μM Csn B for 24 h was analyzed by qPCR (left panel). Jeko cells treated with 10 μM Csn B was used for cell cycle and apoptosis analysis by flow cytometry (right panel). Error bars represent mean ± SD of triplicates (**$p<0.01$, ***$p<0.001$).

Supplementary Figure 3. (A) Cells were treated with/without doxycycline for indicated time points before trypan blue dye exclusion viability assay. Error bars represent mean ± SD of triplicates. (B) Jeko, Maver-1, and Rec-1 cells were treated with 10 μM and 20 μM DIM-C-pPhOH for 6 days, and trypan blue dye exclusion viability assay was then performed. Error bars represent mean ± SD of triplicates. (C) Synergism between DIM-C-pPhOH and ibrutinib in cell killing in Jeko and Rec-1 cells. Cells were treated with ibrutinib and DIM-C-pPhOH 6 days before trypan blue dye exclusion viability assay. Error bars represent mean ± SD of triplicates.

Supplementary Figure 4. (A) Venn diagrams showing the overlap of downregulated or upregulated genes between Jeko and Rec-1 cells after NR4A1 knockout by RNA-seq. (B) GSEA analysis of genes in NR4A1 knockout cells. The gene signature of G2/M checkpoint and PI3K/AKT/mTOR signaling is shown. Heatmaps show G2/M check point or PI3K/AKT/mTOR-associated genes regulated by NR4A1 sgRNAs in Jeko and Rec-1 cells.

Fig. S1

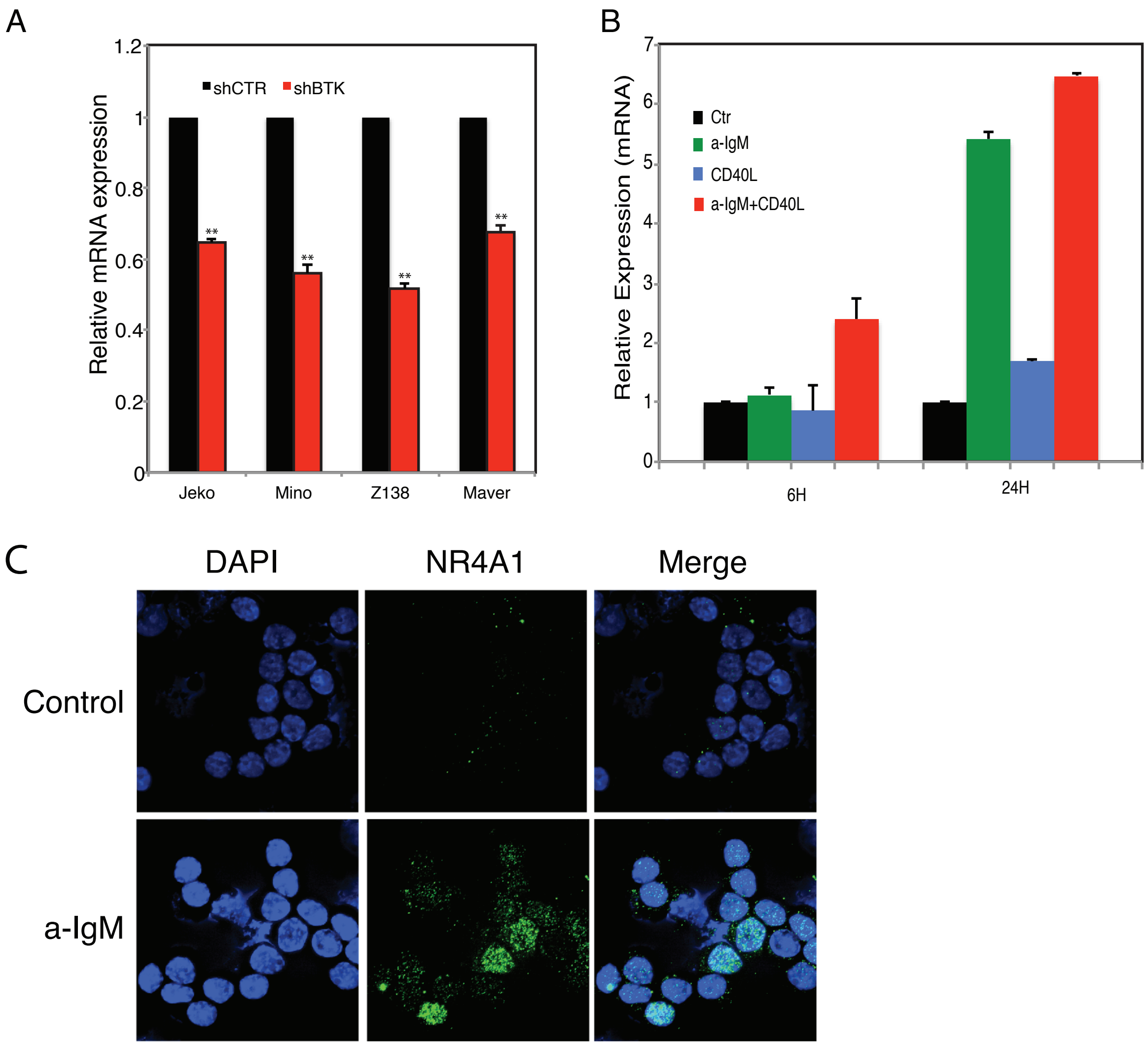
A
1.2
1
0.8
0.6
0.4
0.2
0
Relative mRNA expression
shCTR
shBTK
**
Jeko
Mino
Z138
Maver
B
7
6
5
4
3
2
1
0
Relative Expression (mRNA)
Ctr
a-IgM
CD40L
a-IgM+CD40L
6H
24H
C
DAPI
NR4A1
Merge
Control
a-IgM

Fig. S2

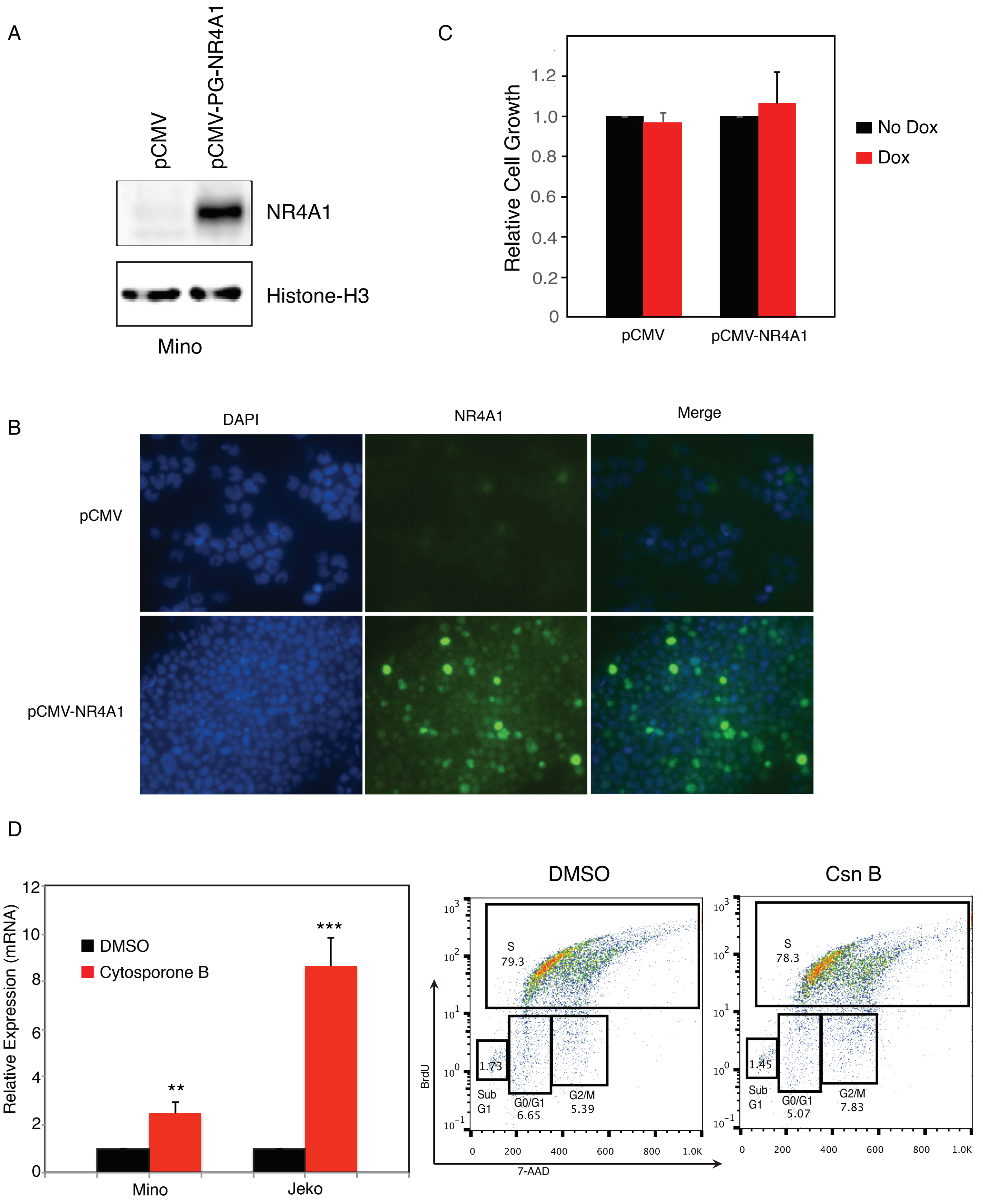

A
pCMV
pCMV-PG-NR4A1
NR4A1
Histone-H3
Mino
C
Relative Cell Growth
0
0.2
0.4
0.6
0.8
1.0
1.2
pCMV
pCMV-NR4A1
No Dox
Dox
B
DAPI
NR4A1
Merge
pCMV
pCMV-NR4A1
D
Relative Expression (mRNA)
0
2
4
6
8
10
12
DMSO
Cytosporone B
**
***
Mino
Jeko
DMSO
Csn B
BrdU
7-AAD
S
79.3
1.73
Sub G1
G0/G1
6.65
G2/M
5.39
S
78.3
1.45
Sub G1
G0/G1
5.07
G2/M
7.83
0
200
400
600
800
1.0K

Fig. S3

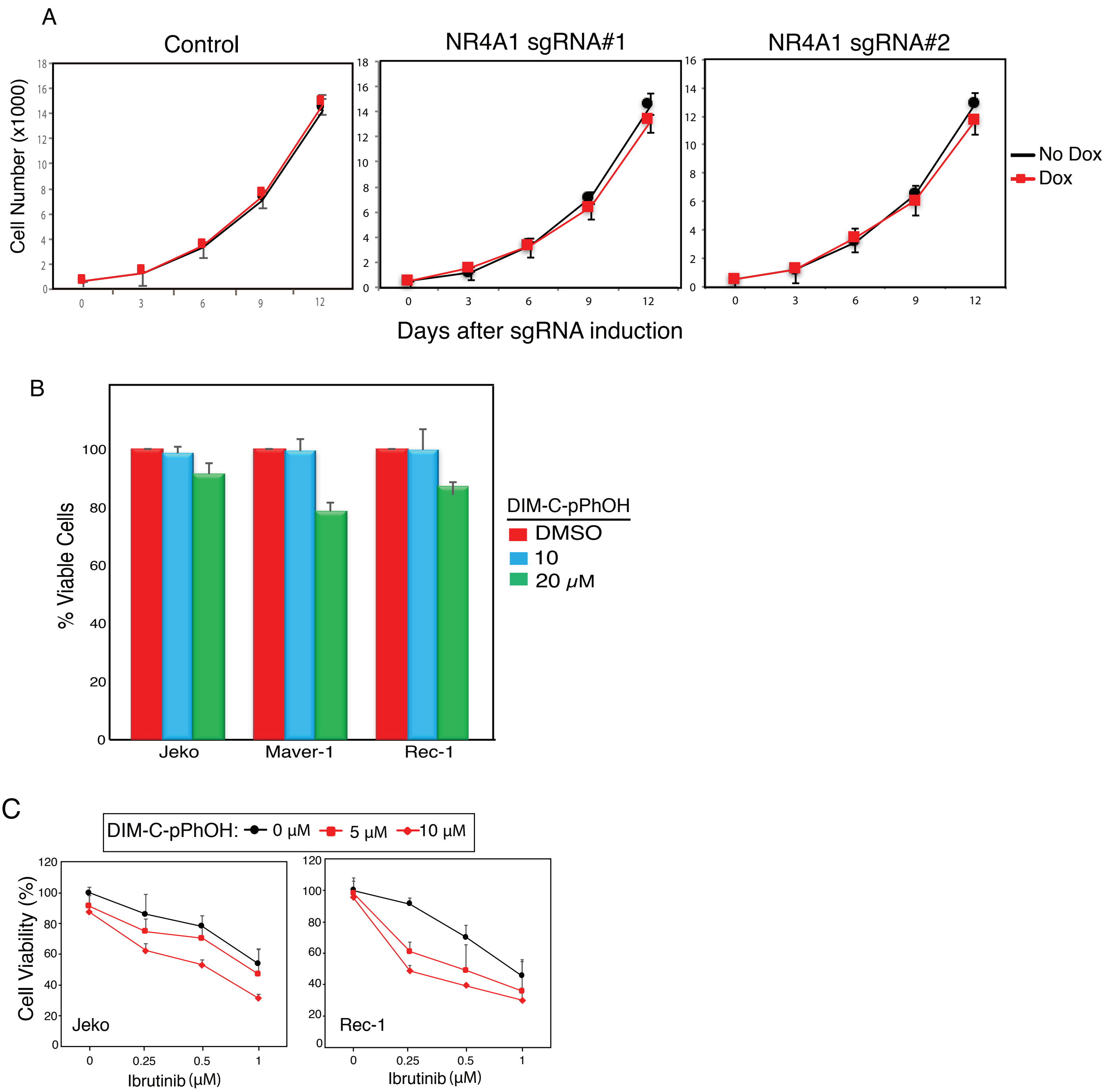
A
Control
NR4A1 sgRNA#1
NR4A1 sgRNA#2
Cell Number (x1000)
Days after sgRNA induction
No Dox
Dox
B
% Viable Cells
Jeko
Maver-1
Rec-1
DIM-C-pPhOH
DMSO
10
20 μM
C
DIM-C-pPhOH: 0 μM 5 μM 10 μM
Cell Viability (%)
Jeko
Rec-1
Ibrutinib (μM)
Ibrutinib (μM)

Fig. S4

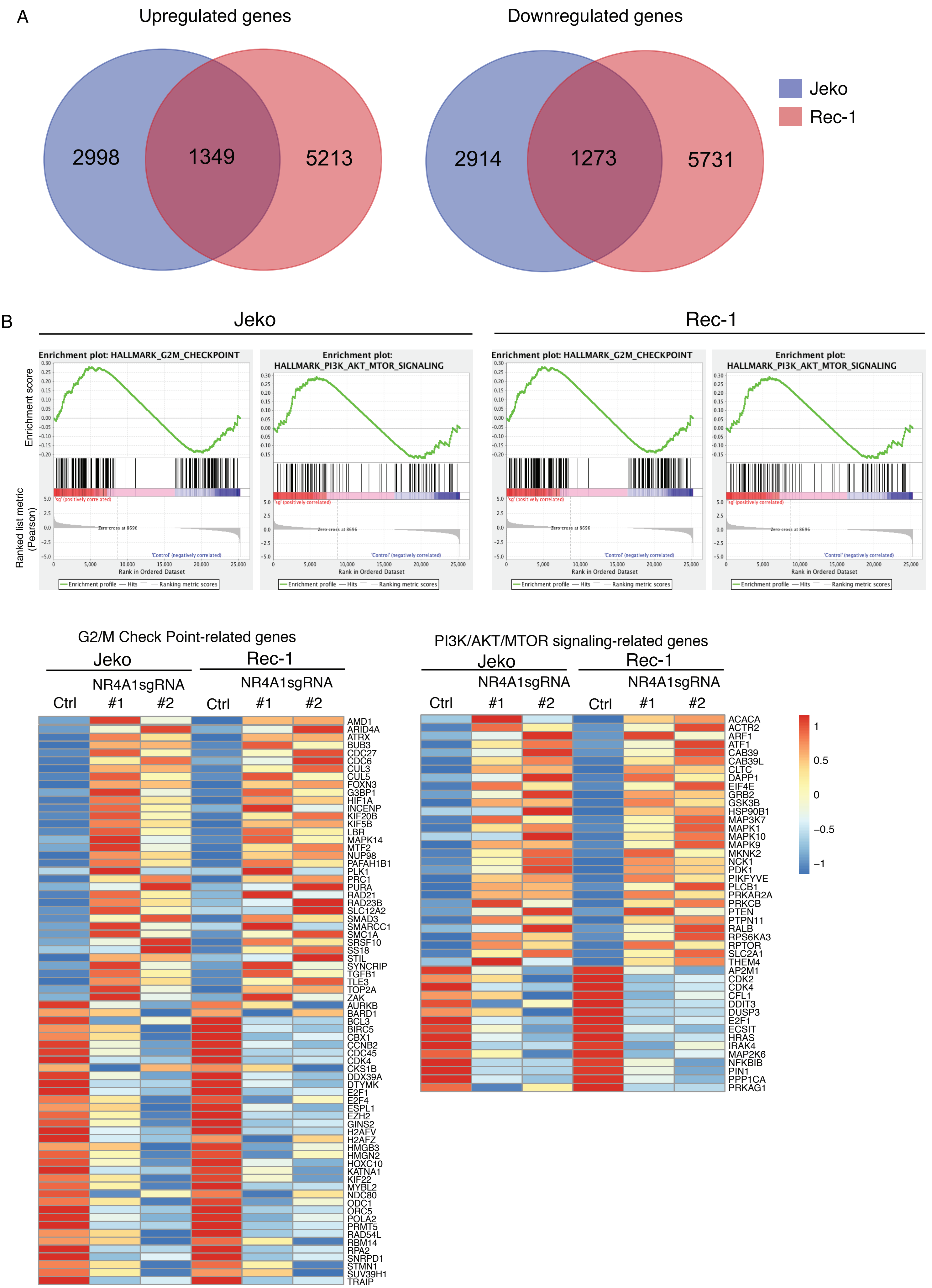
A
Upregulated genes
2998
1349
5213
Downregulated genes
2914
1273
5731
Jeko
Rec-1
B
Jeko
Rec-1
Enrichment plot: HALLMARK_G2M_CHECKPOINT
Enrichment plot: HALLMARK_PI3K_AKT_MTOR_SIGNALING
Enrichment score
Ranked list metric (Pearson)
Rank in Ordered Dataset
G2/M Check Point-related genes
Jeko
Rec-1
NR4A1sgRNA
Ctrl
#1
#2
AMD1
ARID4A
ATRX
BUB3
CDC27
CDC6
CUL3
CUL5
FOXN3
G3BP1
HIF1A
INCENP
KIF20B
KIF5B
LBR
MAPK14
MTF2
NUP98
PAFAH1B1
PLK1
PRC1
PURA
RAD21
RAD23B
SLC12A2
SMAD3
SMARCC1
SMC1A
SRSF10
SS18
STIL
SYNCRIP
TGFB1
TLE3
TOP2A
ZAK
AURKB
BARD1
BCL3
BIRC5
CBX1
CCNB2
CDC45
CDK4
CKS1B
DDX39A
DTYMK
E2F1
E2F4
ESPL1
EZH2
GINS2
H2AFV
H2AFZ
HMGB3
HMGN2
HOXC10
KATNA1
KIF22
MYBL2
NDC80
ODC1
ORC5
POLA2
PRMT5
RAD54L
RBM14
RPA2
SNRPD1
STMN1
SUV39H1
TRAIP
PI3K/AKT/MTOR signaling-related genes
ACACA
ACTR2
ARF1
ATF1
CAB39
CAB39L
CLTC
DAPP1
EIF4E
GRB2
GSK3B
HSP90B1
MAP3K7
MAPK1
MAPK10
MAPK9
MKNK2
NCK1
PDK1
PIKFYVE
PLCB1
PRKAR2A
PRKCB
PTEN
PTPN11
RALB
RPS6KA3
RPTOR
SLC2A1
THEM4
AP2M1
CDK2
CDK4
CFL1
DDIT3
DUSP3
E2F1
ECSIT
HRAS
IRAK4
MAP2K6
NFKBIB
PIN1
PPP1CA
PRKAG1
1
0.5
0
−0.5
−1
